# The use of non-invasive fetal electrocardiography in diagnosing second-degree fetal atrioventricular block

**DOI:** 10.1186/s40748-017-0053-1

**Published:** 2017-08-03

**Authors:** Igor Lakhno, Joachim A. Behar, Julien Oster, Vyacheslav Shulgin, Oleksii Ostras, Fernando Andreotti

**Affiliations:** 1 Department of Perinatology, Obstetrics and Gynecology Kharkiv Medical Academy of Postgraduate Education, 58 Amosova Street, Kharkiv, 61176 Ukraine; 2 Israel Institute of Technology – Technion, Haifa, Israel; 3 INSERM, Nancy, France; 4 National Aerospace University Kharkiv Aviation Institute, Kharkiv, Ukraine; 5 Fetal Cardiology Unit, Ukrainian Children’s Cardiac Center, Kyiv, Ukraine; 6 Institute of Biomedical Engineering, University of Oxford, Oxford, UK

**Keywords:** Fetal monitoring, Non-invasive fetal electrocardiography, Atrioventricular block

## Abstract

**Background:**

Complete atrioventricular block in fetuses is known to be mostly associated with autoimmune disease and can be irreversible if no steroids treatment is provided. Conventional methods used in clinical practice for diagnosing fetal arrhythmia are limited since they do not reflect the primary electrophysiological conduction processes that take place in the myocardium. The non-invasive fetal electrocardiogram has the potential to better support fetal arrhythmias diagnosis through the continuous analysis of the beat to beat variation of the fetal heart rate and morphological analysis of the PQRST complex.

**Case presentation:**

We present two retrospective case reports on which atrioventricular block diagnosis could have been supported by the non-invasive fetal electrocardiogram. The two cases comprised a 22-year-old pregnant woman with the gestational age of 31 weeks and a 25-year-old pregnant woman with the gestational age of 41 weeks. Both women were admitted to the Department of Maternal and Fetal Medicine at the Kyiv and Kharkiv municipal perinatal clinics. Patients were observed using standard fetal monitoring methods as well as the non-invasive fetal electrocardiogram. The non-invasive fetal electrocardiographic recordings were analyzed retrospectively, where it is possible to identify the presence of the atrioventricular block.

**Conclusions:**

This study demonstrates, for the first time, the feasibility of the non-invasive fetal electrocardiogram as a supplementary method to diagnose of the fetal atrioventricular block. Combined with current fetal monitoring techniques, non-invasive fetal electrocardiography could support clinical decisions.

## Background

Fetal arrhythmia (FA) often associated with intrauterine deterioration, which may play an important role in a scenario of fetal demise [1–4]. FA is frequently found as a single disease, but there is an increased risk of comorbidities such as structural malformations of the heart and other organs [5].

Fetal AV block is associated with maternal autoantibodies and fetal structural heart disease in approximately 45–48% of cases and is isolated and of unclear etiology in 4–10% of cases [6]. Complete AV block occurs in approximately 1 out of 20,000 live births [4]. The rate of this type of congenital FA in pregnant women with lupus erythematosus is 1 out of 15,000 live births [7]. The incidence of fetal complete AV block in antibody-positive anti-Ro/SSA and/or anti-La/SSB patients is 1:50 [7]. Such congenital heart defects with left-sided isometrism or transposition of great arteries feature high mortality rates [8].

The AV block is the main responsible for sustained fetal bradycardia. The AV block of the first or second degree can be followed by the spontaneous reestablishment of normal sinus rhythm after delivery [2–4]. However, the second degree of AV block may be a result of the restricted impulse conduction in the case of atrial flutter (AFL) [1, 5]. This type of FA is defined as atrial rate from 300 beats/min to 600 beats/min and fixed or variable AV block with two atrial contractions to every ventricular contraction (2:1). AFL is routinely diagnosed in 1/4 to 1/3 cases of fetal tachyarrhythmia [1, 4, 6].

Conventional methods used in clinical practice for diagnosing FA are limited. The most popular techniques for fetal heart rate monitoring are based on ultrasound reflecting the atrial and ventricular contractions and blood flow, e.g. M-mode, tissue Doppler, hemodynamic Doppler and colored M-mode [1–6]. Such methods are valuable in the assessment of the cardiac anatomy and hemodynamics. However, ultrasound techniques provide an only temporal mapping of mechanical cardiac events and cannot reflect the primary electrophysiological conduction processes happening in the myocardium. Doppler techniques are used to determine the averaged time intervals between atrial and ventricular systoles as well as consecutive heart beats [9]. For instance, cardiotocography (CTG) captures the variability of mechanically detected cardiointervals. Despite being widely used, CTG is limited in its ability to identify FA [10].

There are currently two alternative methods for the antepartum assessment of fetal cardiac electrophysiological characteristics: magnetocardiography (MCG) and non-invasive fetal electrocardiography (NI-FECG). The first method provides a good measure of the PQRST morphology investigation and broadens the diagnostic spectrum of the FA. However, MCG requires a magnetically shielded room and extremely expensive hardware which limits its availability and long-term use [11]. The latter method, i. e. NI-FECG has the potential to better support FA diagnosis through the continuous analysis of the beat to beat variation of the fetal heart rate (FHR) and morphological analysis of the PQRST complex [12, 13]. However, the extraction of the NI-FECG still remains a challenge due to the frequency and temporal overlap between the maternal electrocardiogram and the fetal electrocardiogram which requires advanced signal processing methods to extract the NI-FECG from the abdominal mixture [14, 15]. In addition, a limited number of public databases are currently available [14]. Nevertheless, recent studies [13, 15] suggest that this fetal monitoring technique is effective in monitoring the fetal heart rate and has potential in enabling the extraction of some key ECG morphological parameters such as the ST [13] segment and QT interval [12].

The treatment of AV block depends on its origin and the degree. The presence of fetal hydrops generally complicates the treatment and worsens the outcome [3–5]. Interventions are always initiated in utero for the transplacental therapy. Thus, intra-peritoneal, intra-amniotic, intra-umbilical, intra-muscular or, even, intra-cardiac injections of the antiarrhythmic agents have been described despite the increased risk for the fetus [1, 5]. Corticosteroids are used in case of complete AV block but the efficacy remains controversial. Digoxin, flecainide, sotalol or amiodarone are involved in the management of AFL. Sotalol has the best fetal-to-maternal ratio because of the complete transfer through the placenta. The application of these drugs sometimes should be continued in the postnatal period of life. However, transplacental treatments are ineffective in the case of tricuspid regurgitation, congestive heart failure or fetal hydrops. For those cases, fetal surgery is nowadays available.

In this paper, we are interested in the NI-FECG as a supplementary method to be used in the combination of the cardiotocography and ultrasound technologies for the diagnosis of FA. For this purpose, we show two case studies where NI-FECG provides additional information that could help diagnose AV block. The NI-FECG tracing was obtained from the maternal abdominal wall with the usage of the Cardiolab Babycard equipment (Scientific and research center “KhAI Medica”, Ukraine) [16]. The sampling rate was 1000 Hz. For both cases reported the study protocol was approved by the Bioethics Committee of the Kharkiv Medical Academy of Postgraduate Education (registration number 0105 U002865).

## Case presentation

### Case 1

**Fig. 1:**
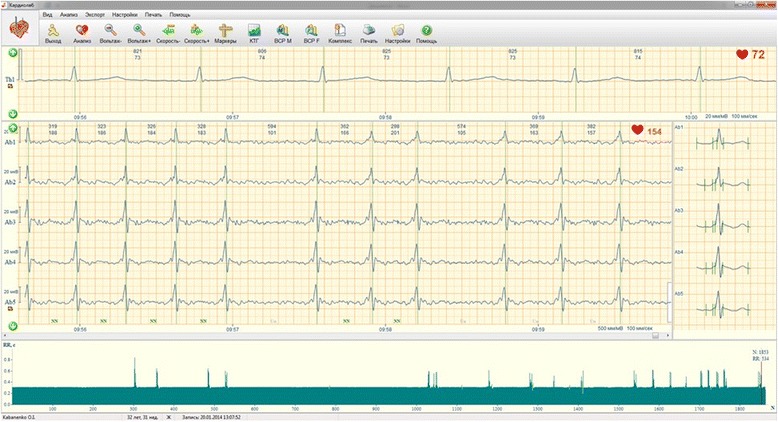
NI-FECG tracing in the Cardiolab Babycard program (case 1). Notice the presence of dropped beats on the NI-FECG channels. These are characteristic patterns of heart block

A 22-year-old pregnant woman with the gestational age of 31 weeks was admitted to the Department of Maternal and Fetal Medicine at the Kyiv municipal perinatal clinics. The cause of the hospitalization was threatened preterm delivery diagnosed by shortened cervix (18 mm on transvaginal ultrasound) and increased uterine tone supported by electrohysterography. The patient was rapidly forwarded to receive tocolysis with hexoprenaline sulfate (25 mg) and betamethasone injections (24 mg totally) for the stimulation of lung maturation. During the ultrasonic investigation, FA was diagnosed. Atrial flutter (AFL) and second-degree AV block with conduction 2:1 were determined during fetal echocardiography. The atrial contractions rate measured with the ultrasonic technique was 353 beats/min and the ventricular rate was 176 beats/min. The fetus was found hydropic but no anatomical abnormalities were visible. The duration of the tocolysis was 48 h. For the investigation of the fetal heart’s electrical activity, NI-FECG was performed. As shown in Fig. 1, the P-wave is not visible on either tracing or on the average fetal complexes. These findings can be characteristic of atrial fibrillation (no P-wave) or AFL (sawtooth P-wave that on the averaged beat cancel each other thus showing no clear P-wave pattern). The drop of multiple cardiac beats is also visible on Fig. 1. Sinusoidal patterns obtained from the RR-time series extracted from the NI-FECG were also found.

The transplacental use of sotalol was started from the moment of diagnosing AFL. The initial regimen was 80 mg 4 times a day (totaling 320 mg per day). The absence of the response to treatment was urged to increase the dosage of sotalol 120 mg twice daily and 80 mg once daily (320 mg daily) and even 80 mg twice daily and 120 mg twice per day (400 mg totally). The trial of transplacental treatment failed within 5 days. The tocolysis failed to stop uterine contractions and the spontaneous preterm labor of a male baby weighing 2100 g was developed by urgent cesarean section. Fetal distress was diagnosed through Apgar score 3 → 5. The newborn was admitted to the neonatal resuscitation unit. The diagnosis of AFL was supported by postnatal ECG. The baby developed multiple organ dysfunction syndrome. The usage of sotalol and amiodarone was not effective and the heart rhythm was irreversible. The baby deceased on the 4th day of life.

### Case 2

**Fig. 2:**
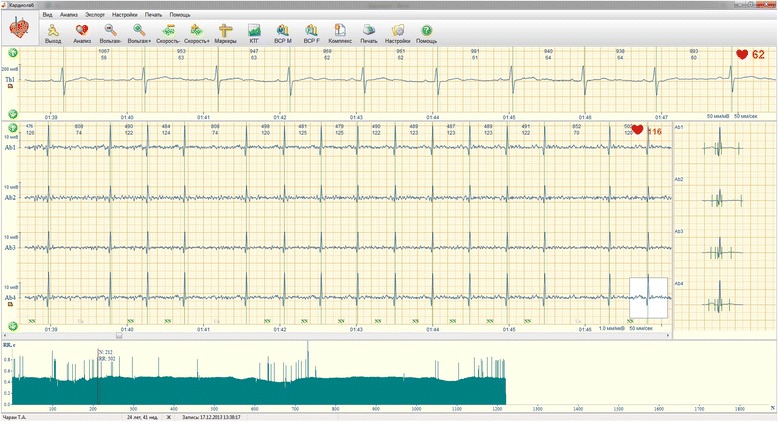
NI-FECG in the case of AV block for a fetus delivered at term (case 2). Notice the dropped beats in the NI-FECG FHR tracings

**Fig. 3:**
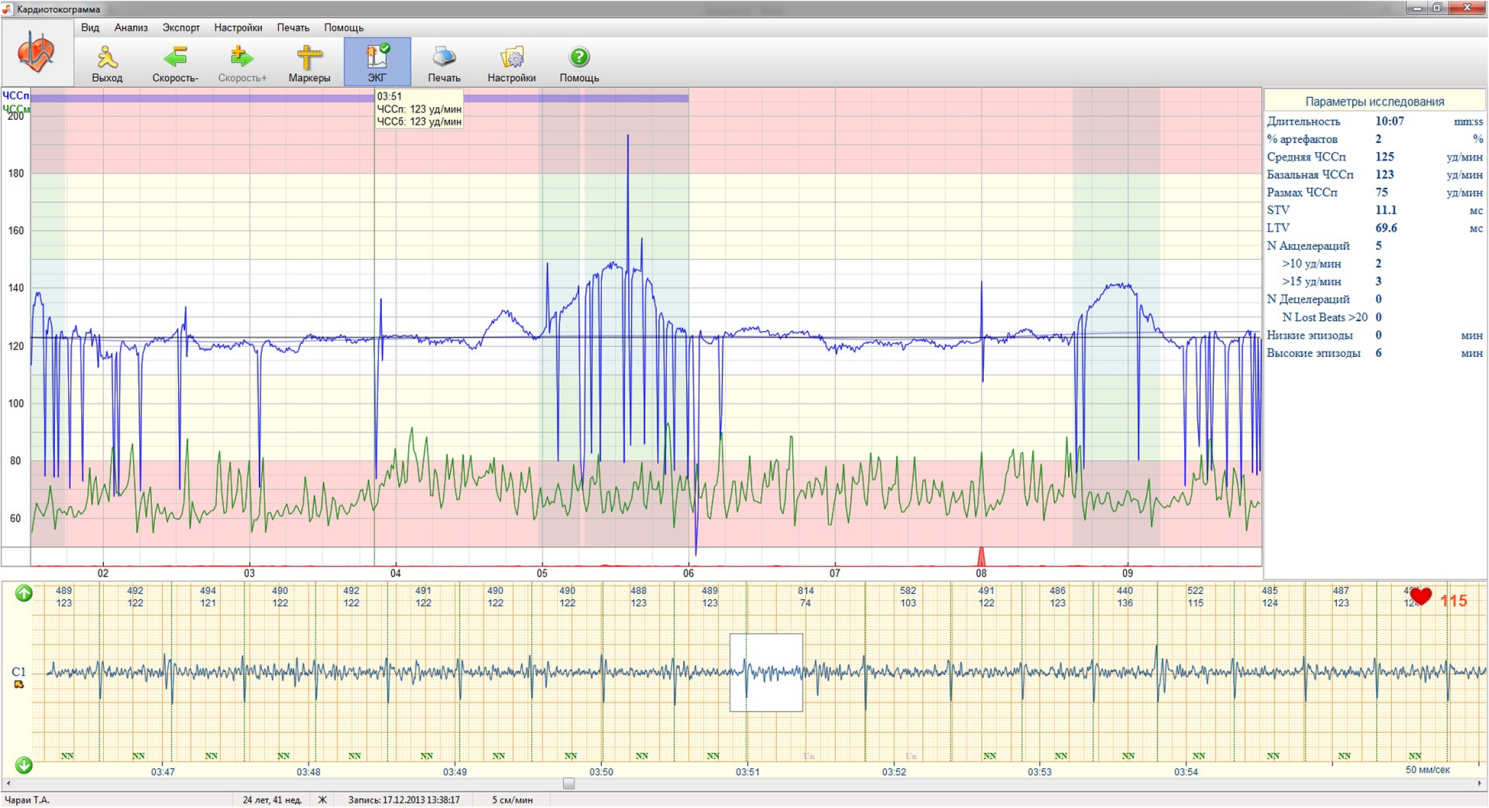
RR time series obtained from NI-FECG recording (case 2) transformed to “beat-to-beat” derived FHR (blue trace). Noticed the presence of multiple dropped beats characterized by local drops in the heart rate

A pregnant woman aged 25 years was admitted to the obstetrical department of Kharkiv Municipal Perinatal clinic with the onset of regular uterine contractions at a term of the 41 weeks. The NI-FECG revealed multiple dropped beats on the ECG (Fig. 2) and on the fetal heart rate (FHR) tracing (Fig. 3).

The delivery occurred without complications, during which continuous electronic fetal monitoring was performed. The average fetal heart rate was higher than 110 beats/min, thus, no antiarrhythmic drugs were not used. A female baby weighing 3750 g with Apgar score 8 → 9 was born. The AV block was reversed to sinus rhythm shortly after birth, confirmed by neonatal ultrasonography and ECG which did not identify any cardiac abnormalities. The newborn was sent home within two days.

## Discussion

Fetal AV block may be suspected in the presence of bradycardia [1–5]. The delayed conduction on AV node is associated with AFL [3–5]. The rate of ventricular systoles in AFL is normal or even increased. Ultrasonic echocardiography is the current standard used for diagnosing FA [2, 3, 6]. Despite its wide usage, M-mode imaging is influenced by fetal position and complexity of the arrhythmia [3]. Therefore, Doppler technique of the pulse-wave assessment in pulmonary vessels is additionally performed [17]. However, the accuracy of such technique in identifying the atrial contractions timing during fetal breathing-like movements, in the case of severe bradycardia and complete AV block may be disturbed.

NI-FECG has the potential to provide good diagnostic information for FA [10, 12–16]. One of the main advantages of the technique is that it enables beat-to-beat measurement of the heart activity versus the classical CTG, which provides an averaged heart rate. We showed, for the first time, the characteristic drop of beats in the NI-FECG enables to identify AV block events on the NI-FECG. Although in the second case reported the AV-block disappeared after birth, in most cases, they persist after birth. Thus, although the two cases presented are the low degree of AV-blocks, they highlight how the NI-FECG technique can be used to spot these events and support diagnosis using this fetal monitoring modality.

The presence of hemodynamic failure (such as fetal hydrops) is an indication for the transplacental treatment even in low degree AV-block. On the other hand, the indication of fetal distress may urge obstetricians to perform cesarean delieveries. Treatment options depend on fetal heart rate, the presence of cardiac defects or fetal hydrops [1–5]. The prognosis is usually much better when cardiac rhythm is reversed in utero [6]. The degree of prematurity also worsens the outcome for the newborn [5, 6]. The FHR sinusoidal pattern observed on the FHR tracing in the first case report reflects the loss of non-linearity in cardiac function, i.e. all cardiointervals became equal. The reason of the irregularity is mostly determined by a failed autonomic control in distressed, anemic or malformed fetuses [10]. A blood test performed after birth confirmed that the baby was indeed anemic.

Previous studies exploring the clinical use of NI-FECG have shown that NI-FECG may provide additional criteria for the diagnosis of fetal distress such as the length of QT interval for diagnosing the long QT syndrome [18]. The NI-FECG has also been used for measuring ST segment changes which can support hypoxia detection during delivery [13, 19]. To date, the commercially FDA/CE marked NI-FECG devices (Monica AN24 (Monica Healthcare, Nottingham, UK) and the Meridian M100/M1000 monitors from MindChild Medical (North Andover, MA) provide accurate FHR measurement. The QT interval length, ST segment changes and detection of specific events such as AV blocks are not readily available on the existing monitors and require further research and clinical validation.

## Conclusions

The presented case reports demonstrate that the NI-FECG has the potential to be used as a supplementary method for the diagnosis of FA. Specifically, we presented two retrospective studies demonstrating the possible usage of the NI-FECG for diagnosing AV block through the analysis of the beat to beat interval obtained by the NI-FECG. In one case the block persisted after birth and in the other case, it disappeared shortly after delivery. Early identification of heart defects using the information provided by the NI-FECG could lead to better management of fetuses and of the newborns.

## Abbreviations

AFL: Atrial flutter
AV: Atrioventricular
CTG: Cardiotocography
FA: Fetal arrhythmia
FHR: Fetal heart rate
MCG: Magnetocardiography
NI-FECG: Non-invasive fetal electrocardiography
